# Improving community participation in clinical and translational research: CTSA Sentinel Network proof of concept study

**DOI:** 10.1017/cts.2020.21

**Published:** 2020-03-10

**Authors:** Deepthi S. Varma, Alvin H. Strelnick, Nancy Bennett, Patricia Piechowski, Sergio Aguilar-Gaxiola, Linda B. Cottler

**Affiliations:** 1 Department of Epidemiology, College of Public Health and Health Professions and College of Medicine, University of Florida, Gainesville, FL, USA; 2 Division of Community Health, Department of Family and Social Medicine, College of Medicine, Albert Einstein, Montefiore Medical Center, Bronx, NY, USA; 3 Department of Medicine, University of Rochester Medical Center School of Medicine and Dentistry, Rochester, NY, USA; 4 Michigan Institute for Clinical and Health Research, University of Michigan, Ann Arbor, MI, USA; 5 Center for Reducing Health Disparities (CRHD), Clinical Internal Medicine, University of California, Davis School of Medicine, Sacramento, CA, USA

**Keywords:** Community health worker (CHW), underrepresented minority, research, referrals, clinical and translational research

## Abstract

**Background:**

Research participation by members of racial or ethnic minority groups continues to be less than optimum resulting in difficulties to generalization of research findings. Community-engaged research that relies on a community health worker (CHW) model has been found effective in building trust in the community, thereby motivating people to participate in health research. The Sentinel Network study aimed at testing the feasibility of utilizing the CHW model to link community members to appropriate health research studies at each of the research sites.

**Methods:**

The study was conducted at six Clinical and Translational Science Award institutions (*N* = 2371) across the country; 733 (30.9%) of the participants were from the University of Florida, 525 (22.0%) were from Washington University in St. Louis, 421 (17.8%) were from the University of California, Davis, 288 (12.1%) were from the University of Michigan, Ann Arbor, 250 (10.5%) were from Rochester, and 154 (6.5%) from Albert Einstein College of Medicine. Trained CHWs from each of these sites conducted regular community outreach where they administered a Health Needs Assessment, provided medical and social referrals, and linked to eligible research studies at each of those sites. A 30-day follow-up assessment was developed to track utilization of services satisfaction with the services and research study participation.

**Results:**

A large majority of people, especially African Americans, expressed willingness to participate in research studies. The top two health concerns reported by participants were hypertension and diabetes.

**Conclusion:**

Findings on the rate of navigation and enrollment in research from this study indicate the effectiveness of a hybrid CHW service and research model of directly engaging community members to encourage people to participate in research.

## Introduction

Participation in research by members of racial or ethnic minority groups and women, older adults, and rural populations continues to be monitored by researchers through ongoing efforts [1–3]. Underrepresentation of these populations in research has occurred, potentially generating findings which cannot be generalized [4–6]. Community-engaged research that relies on a community health worker (CHW) model has been shown to be effective in building trust in the community, thereby motivating people to participate in health research [7–11].

In 2009, the first phase of the Clinical and Translational Science Award (CTSA) Sentinel Network (SN) was funded, consisting of five collaborating CTSA sites (Washington University in St. Louis (WUSTL), University of California at Davis (UC Davis), University of Michigan, University of Rochester, and Albert Einstein) and two partner community organizations (Community-Campus Partnerships for Health and Patient Advocates in Research). HealthStreet, the community-engaged research initiative of the CTSA founded by Dr. Cottler in 1989, formed the basis on which the SN was planned and implemented. More detailed information regarding the conception of the first phase of SN and its results has been published elsewhere and showed how we assess, in real time, concerns and needs of underrepresented populations in order to give people a voice in research [3].

The SN Phase I found that historically underrepresented community members were *more* interested in taking part in health research and oftentimes were willing to participate in a research study for *less* compensation than their counterparts [3,12,13]. Based on these findings, a Phase II was implemented to (a) develop procedures to include informed consent to link community members to appropriate health research studies in sites that had not previously been doing so, (b) increase the thoroughness of community health evaluations and research, (c) detect emerging community issues regarding participation of underrepresented populations in health research, (d) build capacity for CHWs and expand their role in research, and (e) build connections with the community. This paper, a proof of concept, reports on the expanded SN Phase II, which not only assessed the health needs and concerns of community members and connected them to relevant medical and social services and health research opportunities but also followed up participants for 30 days to assess research enrollment status, service utilization, and satisfaction with services referred.

## Materials and Methods

The SN built the capacity of CHWs to engage individuals within their own communities by discussing their health concerns and priorities, past research experiences, and current expectations and linked them to potential research participation opportunities. The first phase of SN included five CTSA institutions across the country: Washington University in St. Louis, St. Louis (WUSTL); University of Rochester, Rochester, New York (Rochester); University of Michigan, Ann Arbor (U-M); Albert Einstein College of Medicine, Bronx, New York; and UC Davis. In 2012, a Phase II of SN was implemented and added the University of Florida (UF), where the Principal Investigator (Dr. Cottler) relocated. SN sites were geographically and demographically diverse. By the conclusion of Phase II recruitment, the SN had assessed over 8000 community members across the country, utilizing the CHW model to assess community members’ actual medical conditions and concerns in real time and to link them to social and medical services and opportunities to participate in health research within their own communities.

### Assessments

The Health Intake assessment for SN Phase II was scaled up from that of the SN Phase I assessment [3]; it assessed head-to-toe health conditions over the lifetime (e.g., arthritis, asthma, cancer, depression, diabetes, muscle and bone, heart conditions, blood pressure, and kidney diseases). During the course of the intake, CHWs asked each respondent for at least two means of contact, such as a cell/home/business phone number, email address, and a physical mailing address. The intake was vigorously pilot tested by all sites before it was used. All baseline assessments were done in a face-to-face interview by the CHW.

A 30-day follow-up assessment by phone was developed to track utilization of services to which each participant was referred during the initial Health Intake assessment. In addition, participants were asked if they sought any medical or social services on their own within the past 30 days. If the respondent reported obtaining a service referral or self-referral, satisfaction with the service obtained was assessed. Conversely, if a respondent reported not obtaining a service, the reason for such was elicited. Lastly, participants who were linked to potential research opportunities were asked if they were contacted by the corresponding research study coordinator and whether they were enrolled into the study.

### CHW Recruitment and Training

CHWs underwent rigorous training for all assessments and 30-day follow-up protocols. Biweekly meetings were held with all participating sites to monitor data collection procedures and ensure fidelity to protocols. CHWs approached community members off-campus (i.e., “in the community”) at barbershops, laundromats, bus stops, community agencies, churches, parks, health care facilities, and other sites. Each site obtained institutional review board approval from their corresponding institution. After clearly describing the study, CHWs obtained the informed consent, then conducted the Health Intake assessment, and where appropriate, made referrals.

### Data Management and Analysis

All Health Intakes were scanned and sent to WUSTL (lead site but later to UF) in an encrypted, password-protected format. Data were entered using REDCap (Research Electronic Data Capture) software [14]. Data Coordinators at participating sites collaborated with WUSTL to obtain a security certificate and REDCap user account by which to access and view the data. Data entry and quality control were performed on a regular basis to ensure protocol fidelity.

Data were analyzed for site and race/ethnicity using SAS version 9.2 software (SAS Institute, Cary, NC, USA). Means and standard deviations were calculated for continuous variables, and binomial proportions reported with corresponding confidence intervals for categorical variables.

## Results

### Demographic Characteristics

Table 1 shows the demographic characteristics of SN Phase II participants by site. Overall, a total of 2371 participants were assessed across the six collaborating sites: 733 (30.9%) of the participants were from UF, 525 (22.0%) were from WUSTL, 421 (17.8%) were from UC Davis, 288 (12.1%) were from U-M, 250 (10.5%) were from Rochester, and 154 (6.5%) from Albert Einstein College of Medicine. The sample in Phase II was ethnically diverse with the majority of respondents reporting Black or African-American race (53.1%), followed by White (25.2%) and Hispanic/Latino ethnicity (14.1%). Frequency of “Other” races, such as Asian (2.1%), American Indian/Alaska native (1.1%), and others (3.9%), was low. Women comprised a majority (54.8%) of the Phase II sample, regardless of site, with the exception of Rochester. The mean age of the sample was 43 years (standard deviation ±14.9); Phase II participants tended to have a high-school diploma (a mean of 12.8 years of education ±2.6), and an average body mass index of 29.1 (±7.1).

**Table 1.** Demographic characteristics of Sentinel Network phase II participants by site

	University of Florida (*N* = 733)		Washington University in St. Louis (*N* = 525)		University of California, Davis (*N* = 421)		University of Michigan, Ann Arbor (*N* = 288)		University of Rochester, Rochester, New York (*N* = 250)		Albert Einstein College of Medicine, Bronx, New York (*N* = 154)		All sentinel (*N* = 2371)	
	*N*	%	*N*	%	*N*	%	*N*	%	*N*	%	*N*	%	*N*	%
Female	407	55.5	265	50.5	301	71.7	178	62.2	53	21.2	93	60.4	1297	54.8
Race: American Indian/Indian/Alaskan Native	1	0.1	5	1.0	11	2.6	4	1.4	5	2.0	0	0.0	26	1.1
Asian	13	1.8	1	0.2	19	4.5	13	4.5	1	0.4	3	1.9	50	2.1
Black or African American	456	62.2	408	77.9	125	29.7	111	38.7	110	44.0	48	31.2	1258	53.1
Native Hawaiian/Pacific Islander	1	0.1	0	0.0	12	2.9	0	0.0	1	0.4	0	0.0	14	0.6
White	194	26.5	80	15.3	92	21.9	126	43.9	100	40.0	4	2.6	596	25.2
Hispanic	46	6.3	14	2.7	142	33.7	11	3.8	25	10.0	95	61.7	333	14.1
Other	22	3.0	16	3.1	20	4.8	22	7.7	8	3.2	4	2.6	92	3.9
Age (mean (±SD))	40 (±15.3)		41 (±13.1)		47 (±15.8)	42 (±15.7)		46 (±11.0)		44 (±16.1)		43 (±14.9)		
Years of education (mean (±SD))	12.8 (±2.6)		12.5 (±2.1)		12.7 (±2.7)	14.2 (±2.5)		11.9 (±2.2)		13.0 (±3.1)		12.8 (±2.6)		
Body mass index (mean (±SD))	29.0 (±6.9)		29.4 (±7.4)		29.7 (±7.3)	28.5 (±7.2)		28.5 (±6.7)		29.4 (±6.2)		29.1 (±7.1)		

SD, standard deviation.

### Health Concerns of Participants

Shown in Table 2 are the top five health concerns among those with a concern. The top concerns were heart problems, hypertension, and diabetes. Hypertension was among the top health concerns among all but White participants. Cancer was reported by all races as a top health concern except for Asians. Concerns regarding “weight” were reported among the top five health concerns for all. Mental health was the second most mentioned concern among White participants.

**Table 2.** Top five health concerns of participants by race/ethnicity (among those with a concern)

Health concerns	White (*n* = 550)	African American (*n* = 1136)	Hispanic/Latino (*n* = 303)	Asian (*n* = 44)	Other (*n* = 117)
1st	Heart problems	Hypertension	Diabetes	Diabetes	Hypertension
2nd	Mental health	Diabetes	Cancer	Hypertension	Diabetes
3rd	Cancer	Heart	Hypertension	Staying healthy/healthy lifestyle	Weight
4th	Weight	Weight	Heart	Weight	Cancer
5th	Diabetes	Cancer	Weight	Heart	Mental health

### Health Conditions

Table 3 shows the health conditions that participants reported in the Health Intakes regarding their own health. Diseases and symptoms of the muscles and bones were reported at the highest rate among all five racial and ethnic groups (i.e., “Others” (51.9%), Whites (49.2%), Hispanic/Latinos (48%), and African Americans (44.0%), and Asians (29.2%)). Conditions that were next most relevant were depression (Whites), high blood pressure (African Americans), heart conditions (Hispanics), and arthritis (other race). Asians reported both muscle and bone and heart conditions as their most common condition but at a rate which was considerably lower than the other four groups.).

**Table 3.** Health conditions reported by Sentinel Network Phase II participants, by race/ethnicity: 2012–2013 (*n* = 2371)^a^

Characteristics	White (*n* = 594)	African American (*n* = 1255)	Hispanic/Latino (*n* = 332)	Asian (*n* = 48)	Other (*n* = 131)
	*n* (%)	*n* (%)	*n* (%)	*n* (%)	*n* (%)
Arthritis	192 (32.6)	337 (27.5)	78 (24.2)	13 (27.7)	48 (37.5)
Asthma	124 (20.9)	226 (18.1)	66 (20.0)	3 (6.3)	34 (26.2)
Cancer	54 (9.1)	50 (4.0)	17 (5.1)	2 (4.2)	10 (7.6)
Depression	227 (38.2)	300 (23.9)	64 (19.5)	4 (8.3)	32 (24.4)
Diabetes	55 (9.3)	161 (13.0)	41 (12.5)	6 (12.5)	19 (15.0)
Muscle and bone	292 (49.2)	549 (44.0)	157 (48.0)	14 (29.2)	68 (51.9)
Heart conditions	218 (37.10	389 (31.6)	88 (27.5)	14 (29.2)	43 (33.3)
High blood pressure	141 (23.7)	425 (33.9)	66 (19.9)	11 (22.9)	36 (27.5)
Kidney	143 (24.1)	193 (15.5)	63 (19.9)	5 (10.4)	29 (22.7)
Smoked cigarettes in past 30 days	234 (39.6)	608 (48.7)	90 (28.3)	4 (8.5)	60 (46.2)

a *N* = 11 participants with missing race/ethnicity information.

### Research Experiences and Perceptions

**Fig. 1.**
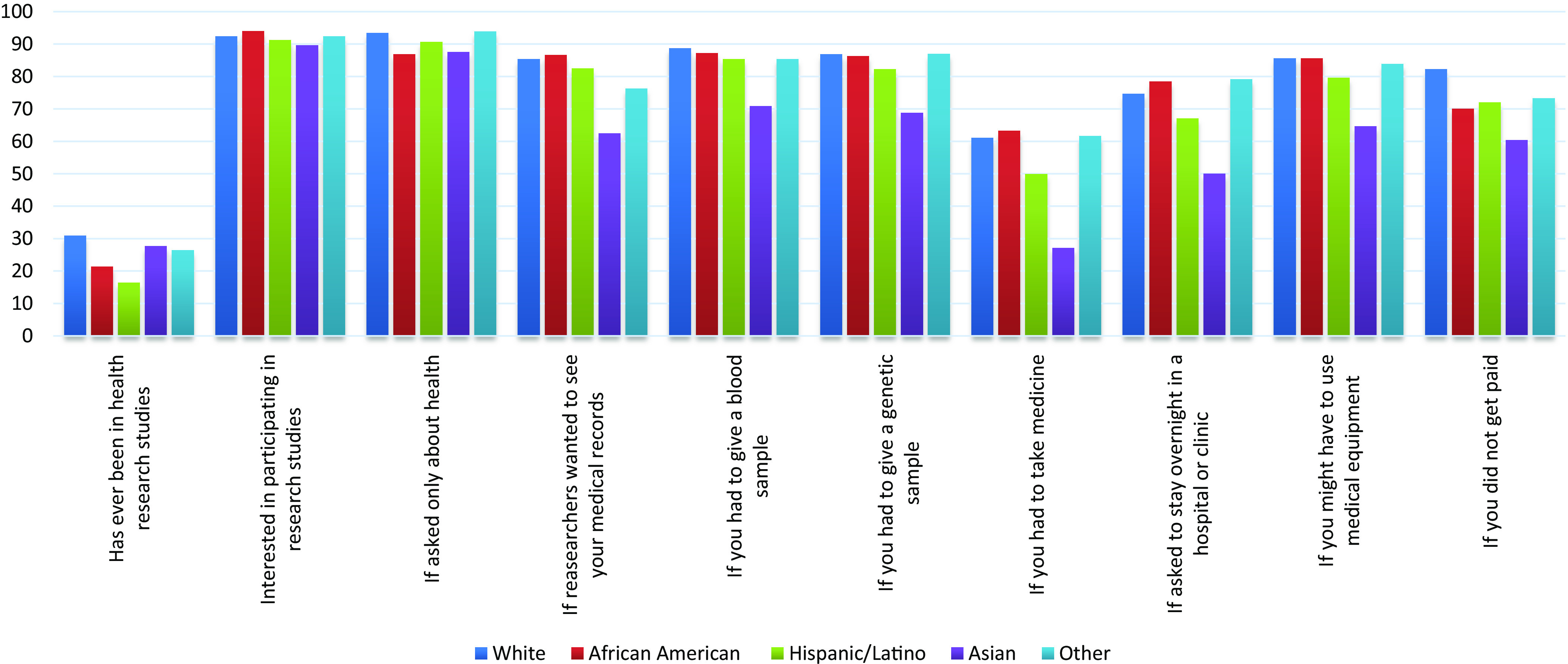
Research experience and perceptions reported by Sentinel Network Phase II participants, by race/ethnicity: 2012–2013 (*n* = 2371)^a^. ^a^ *N* = 11 participants with missing race/ethnicity information.

Findings on participant’s research experiences and perceptions (Fig. 1) revealed that a large majority (89.6%–94.0%) expressed considerable interest in participating in a health research study (i.e., “definitely” and “maybe”). While only 17.1% reported participating in health research in Phase I, 23.5% of Phase II participants reported participation. African Americans expressed slightly higher rates of interest in participation (94.0% vs 91% in Phase I) compared to Whites and other races (both 92.4%), and Latinos (91.2%) with Asians having the lowest rate of interest in research participation (89.6%). More Whites (82.3%) agreed to participate in research without any payment compared to all other participants. All participants except for Asians reported high willingness to participate in a study with a blood sample for genetic studies. Participants mentioned mean $87.46 as a fair amount of remuneration to participate in a study that lasted 1.5 hours with a blood sample compared to $73.54 reported by Phase I participants. Across different ethnicities, the highest mean expected compensation was reported by African Americans ($105.88 ± $173.97) and the least by Whites ($48.36 + $74.95).

When asked about what types of studies they would be willing to participate in, participants reported the highest willingness to participate in a study that only asked about their health (86.7%–93.9%). Having to take medicine was the least likely to hold the interest of the participants with a willingness rate ranging from 27.1% (Asians) to 61.7% (“Other”).

### Service Referrals

Newly implemented in Phase II was utilization of medical and social service referrals provided to study participants (Table 4). Overall, 65.3% of all participants were referred by the CHW to at least one social or medical service for a total of about 4000 service referrals made. 61.6% of the participants were contacted for 30 days for a follow-up survey assessing utilization of and satisfaction with services to which they were referred. Follow-up rates range from a high of 75.7% (UF) to a low of 39% (Einstein).

**Table 4.** Referrals at baseline and 30-day follow-up among total participants (*n* = 2371 people)

Participants	University of Florida (*N* = 733)	Washington University in St. Louis (*N* = 525)	University of California, Davis (*N* = 421)	University of Michigan, Ann Arbor (*N* = 288)	University of Rochester, Rochester, New York (*N* = 250)	Albert Einstein College of Medicine, Bronx, New York (*N* = 154)	All sites (*N* = 2371)
Participants referred to at least one service at baseline assessment (% of participants)	493 (67.3%)	463 (88.2%)	236 (56.1%)	204 (70.8%)	124 (49.6%)	28 (18.2%)	1548 (65.3%)
Participants successfully contacted for 30-day follow-up (% of participants)	555 (75.7%)	326 (62.1%)	215 (51.1%)	185 (64.2%)	120 (48.0%)	60 (39.0%)	1461 (61.6%)
Service referral events							
Total *service referrals* given at baseline assessment	1093	1430	490	451	194	40	3698
Total service referrals reported at 30-day follow-up (by those who were successfully contacted at 30-day follow-up) (% of total service referrals)	677 (61.9%)	836 (58.5%)	258 (52.7%)	309 (68.5%)	79 (40.7%)	15 (37.5%)	2174 (58.8%)
Study referral events							
Total study referrals given at assessment	531	354	123	284	247	80	1619
Study referrals reported at 30-day follow-up (by those who were successfully contacted at 30-day follow-up) (% of total study referrals)	340 (64.0%)	201 (56.8%)	75 (61.0%)	182 (64.1%)	117 (47.4%)	40 (50.0%)	955 (59.0%)

### Follow-up at 30 Days

Among 2174 social service referrals given at all sites, 386 (17.8%) were reported as obtained over a 30-day period (Table 5). Of note, among these services obtained, 91.2% were reported as “helpful” to the respondent; 94.8% of participants reported high satisfaction.

**Table 5.** Rate of referral utilization reported at 30-day follow-up (*N* = 2174 referral events)

Followed up at 30-day post-baseline	University of Florida (*N* = 677)	Washington University in St. Louis (*N* = 836)	University of California, Davis (*N* = 258)	University of Michigan, Ann Arbor (*N* = 309)	University of Rochester, Rochester, New York (*N* = 79)	Albert Einstein College of Medicine, Bronx, New York (*N* = 15)	All sites (*N* = 2174)
Obtained services by 30-day follow-up (%)	101 (14.9%)	187 (22.4%)	21 (08.1%)	66 (21.4%)	8 (10.1%)	3 (20.0%)	386 (17.8%)
Referral services reported as helpful (%)	93 (92.1%)	165 (88.2%)	19 (90.5%	65 (98.5%	8 (100.0%)	2 (66.7%)	352 (91.2%)
Satisfied with the obtained services (%)	94 (93.1%)	177 (94.7%)	21 (100.0%)	64 (97.0%)	7 (87.5%)	3 (100.0%)	366 (94.8%)

### Study Referrals and Enrollment

A total of 1619 participants from all sites were given a research study referral at the baseline (Table 6). We were able to contact 955 (59%) out of 1619 participants for a 30-day follow-up; 355 participants (37.2%) reported that they had been contacted by the study coordinator. Table 6 also shows the rate of enrollment of participants into an eligible study. At the 30-day follow-up, 42.3% of participants contacted by a study coordinator reported that they were enrolled in a research study. Across collaborating sites, UF had the highest rate of study referrals for the participants (64%); U-M had the highest rate of 30-day follow-up (64.1%); and WUSTL reported the highest percent of study coordinator contacts (66.7%) and of study enrollments (50.7%) compared with other sites. Four of five participants enrolled in a study (80.7%) reported satisfaction regarding the navigation and linking process to research studies at their respective universities, but with only one study enrolled at both Einstein and Rochester and none at UC Davis, these rates of satisfaction are limited in their generalizability even to the six Sentinel sites.

**Table 6.** Rate of research study enrollment by the participants at 30-day follow-up

Participants	University of Florida (*N* = 733)	Washington University in St. Louis (*N* = 525)	University of California, Davis (*N* = 421)	University of Michigan, Ann Arbor (*N* = 288)	University of Rochester, Rochester, New York (*N* = 250)	Albert Einstein College of Medicine, Bronx, New York (*N* = 154)	All sites (*N* = 2371)
Total number of participants who were given a study referral at baseline	531 (72.4%)	354 (67.4%)	123 (29.2%)	284 (98.6%)	247 (98.8%)	80 (51.9%)	1619 (68.3%)
Total participants who received a study referral being contacted at 30-day follow-up (%)	340 (64.0%)	201 (56.8%)	75 (61.0%)	182 (64.1%)	117 (47.4%)	40 (50.0%)	955 (59.0%)
No. of participants reported making contact with study coordinator (%)	112 (32.9%)	134 (66.7%)	9 (12.0%)	84 (46.2%)	11 (09.4%)	5 (12.5%)	355 (37.2%)
Among those made a contact, number of participants enrolled in a study by the 30-day follow-up (%)	43 (38.4%)	68 (50.7%)	0 (0%)	37 (44.0%)	1 (09.1%)	1 (20.0%)	150 (42.3%)
Satisfied with study navigation and linking process (%)	29 (67.4%)	60 (88.2%)	0 (0%)	31 (83.8%)	0 (00.0%)	1 (100.0%)	121 (80.7%)

## Discussion

The goal of the second phase of the SN study was to assess the impact of CHWs offering social service and research study referrals to racial and ethnic group members who are underrepresented in clinical and translational research and to strengthen the ongoing collaboration across the six CTSA sites.

Findings from this study show that a large majority of people, especially African Americans, express willingness to participate in research studies. This highlights a paradigm shift from historical mistrust toward research and researchers toward a more positive attitude [15–20]. The wide gap seen in this study between those who expressed an interest in participating in a health research study (93%) and those who have actually participated in any type of research study (23.5%), whether clinical- or community-based research, indicates that there are a large number of community members who are willing to participate in research studies but do not get to a study. Many reasons could be cited for this: lack of diversity in staff, ineligible, no studies for their condition, and/ or bias on the part of the coordinator. This is similar to previous findings reported regarding the willingness to participate [21,22]. Moreover, the findings also show that though they are interested in research, the participants clearly distinguish the types of research in which they would be willing to participate. Successful enrollment of 42% of the participants reinforces the previous findings that willingness of community members to participate in research can be translated to actual enrollment by contacting them directly and navigating them to eligible studies [23–25]. The fair compensation mentioned by participants in this study is higher than what most studies currently provide. This shows the expectation of the community members. It also shows the importance of reimbursing community members adequately and appropriately not only to increase research study enrollment and retention but also to improve the community-academic relationships.

The top two health concerns reported by participants – hypertension and diabetes – remained the same in both Phase I and II, highlighting the salience of these conditions in people’s minds as well as the public health significance of screening individuals regularly for these conditions [26]. This finding is especially significant since the majority of the participants of this study (53.1%) were Black or African American, and previous studies have consistently indicated a higher prevalence of these two conditions among them compared to Whites and other ethnic groups in the USA [27–29]. Further, this also indicates the increased awareness regarding chronic diseases among the public, especially among African Americans.

Over one-third of the participants reported existing health conditions were muscle and bone diseases, heart conditions, arthritis, and mental health conditions such as depression/bipolar disorder. Further, 34% of African Americans reported high blood pressure as the second top health condition followed by heart conditions (32%). As reported in previous studies, smoking continues to be one of the strongest risk factors for noncommunicable diseases among African Americans with 48.7% of the participants reporting smoking a cigarette in the past 30 days [30,31]. This is much higher than the current cigarette smoking rate of 15.1% among adults in the USA [32].

Findings from this study highlighted the continuing disparity in access to medical care that exists among different race/ethnicity groups. Phase II showed African Americans reporting the lowest proportion (64.3%) having any medical insurance, which is a shift from what we observed in Phase I, where the Hispanic/Latino community had the lowest proportion with medical insurance. Findings from this study also support the previous research on benefits of utilizing the CHW model to improve access to care and research participation [33]. Increased referral rates indicate that with adequate screening, CHWs could facilitate medical and social service referrals to local resources that are easily accessible and affordable for community members. However, the low referral utilization rate shows the various barriers that are personal and systemic. It also highlights the need for additional follow-ups by CHWs, provision of free or low-cost transportation to improve access to services, and reminders to ensure the utilization of the provided referrals to community members. However, it is encouraging to see that those who utilized the provided referrals reported highest level of satisfaction with the services obtained. We acknowledge that the lack of data on the types of barriers to utilization of the referrals provided is one of the limitations of this study.

## Limitations and Strengths

This was a cross sectional study and was conducted using a convenience sampling strategy. However, we were able to include culturally and ethnically diverse sample by conducting the study at different sites that offsets the limitations due to convenience sampling. Another important strength of this study is that it proved the feasibility of a new model utilizing CHWs in building academic-community partnerships, thereby increasing the participation of all community members in health research studies.

## Conclusion

Building on Phase I of the SN study, we deployed a hybrid model for CHWs building relationships by learning about community members’ health concerns and making both social service and research study referrals. Those referrals were evaluated by a 30-day follow-up assessment of the success of community members receiving those social services and their satisfaction with them. We also assessed the rate they were contacted by research study coordinators and actually enrolled in clinical research studies. One might describe this as a hybrid model of combined social service and research navigation by CHWs. Findings based on the rate of navigation and enrollment in research from this study indicate that the hybrid CHW service and research model of directly engaging community members and encouraging people to participate in research is effective. Phase II of the SN may be considered a “proof of concept” for the hybrid CHW model that better integrates service and research in order to recruit more diverse research volunteers and those underrepresented in our current human research studies.
